# Draft Genome Sequence of 24570, the Type Strain of *Shigella flexneri*

**DOI:** 10.1128/genomeA.00393-15

**Published:** 2015-05-28

**Authors:** Kate S. Baker, Julian Parkhill, Nicholas R. Thomson

**Affiliations:** a Wellcome Trust Sanger Institute, Hinxton, Cambridgeshire, United Kingdom; b London School of Hygiene and Tropical Medicine, London, United Kingdom

## Abstract

*Shigella flexneri* is a diarrheal pathogen that causes a large disease burden worldwide. We sequenced the genome of the publicly available type strain (*S. flexneri* 2a strain 24570) of this bacterial species to increase its utility as a reference. We present genome assembly results and comparisons with other reference strains.

## GENOME ANNOUNCEMENT

*Shigella flexneri* is a highly contagious bacterial pathogen responsible for a large global disease burden. Every year, approximately 750,000 children under the age of 5 years die from diarrheal disease in developing nations (1). Within that demographic group, *Shigella* is one of the top four attributable causes of diarrheal disease (2), with *Shigella flexneri* causing the majority of the shigellosis burden in these areas (3). Bacterial type strains serve as a benchmarking tool for diagnostic and research laboratories around the world (4) and the type strain of the *Shigella flexneri* strain (strain 24570) is available at the American Type Culture Collection.

Genomic DNA from the *Shigella flexneri* type strain 24570 (ATCC 29903) was obtained and sequenced using 150 bp paired-end Illumina (MiSeq) sequencing according to in-house protocols (5, 6), with an approximately 500 bp insert size. Reads (*n* = 2,037,336) were assembled into 327 contiguous sequence scaffolds using Velvet Optimizer (7) and these were annotated using Prokka (8), to produce a high-quality draft genome (9). BLAST comparisons were made to determine the presence/absence of genes in the annotated assembly, and phylogenetic analysis was performed as in reference (10).

The reads of the strain 24570 draft genome mapped against reference genome 2457T (11) with approximately 70× coverage. The assembly had an *N*_50_ of 39,568 bp and a total length of 4.7 MB. Automated annotation predicted the presence of 4,653 coding sequences, including the serotype conferring *gtrII-*gene. Phylogenetic sequence analysis (performed as in reference 10) confirmed that the type strain of *Shigella flexneri* fits within a serotype 2a lineage (not shown). When compared with complete *Shigella flexneri* serotype 2a genomes, 24570 had 1,103 single nucleotide polymorphisms (SNPs) relative to first completed reference genome strain 301 (12), 203 SNPs relative to the common laboratory strain 2457T (11), and 414 SNPs relative to the World War 1 strain, NCTC1 (10). The availability of a draft genome for this publicly available type strain increases its utility as a resource for diagnostic and research laboratories.

### Nucleotide sequence accession numbers.

This whole-genome shotgun project has been deposited in the European Nucleotide Archive under Bioproject number PRJEB2976, sample ERS574920, run ERR738429. Contiguous sequences of the *de novo* assembly have also been deposited at the European Nucleotide Archive under the accession numbers CELV01000001 to CELV01000327.
